# Does Rotation and Anterior Translation Persist as Residual Instability in the Knee after Anterior Cruciate Ligament Reconstruction? (Evaluation of Coronal Lateral Collateral Ligament Sign, Tibial Rotation, and Translation Measurements in Postoperative MRI)

**DOI:** 10.3390/medicina59111930

**Published:** 2023-10-31

**Authors:** Yavuz Selim Karatekin, Harun Altınayak, Lokman Kehribar, Ali Kerim Yılmaz, Esra Korkmaz, Berna Anıl

**Affiliations:** 1 Department of Orthopaedics and Traumatology, Samsun Education and Research Hospital, 55090 Samsun, Turkey; harun240507@gmail.com; 2 Medical Faculty, Department of Orthopaedics and Traumatology, Samsun University, 55090 Samsun, Turkey; lokmankehribar@gmail.com; 3 Faculty of Yasar Doğu Sport Sciences, Ondokuz Mayıs University, 55090 Samsun, Turkey; akerim.yilmaz@omu.edu.tr (A.K.Y.); esrkmz@gmail.com (E.K.); bernahltck@gmail.com (B.A.)

**Keywords:** anterior cruciate ligament, ACL reconstruction, internal rotation, anterior translation, coronal LCL sign, static instability

## Abstract

*Purpose:* The aim of this study was to evaluate the presence of residual instability in the knee after ACL reconstruction through the analysis of MRI findings. *Methods:* This study included patients who underwent isolated ACL reconstruction between December 2019 and December 2021, and had preoperative and postoperative MRI, clinical scores, and postoperative isokinetic measurements. The anterior tibial translation (ATT) distance, coronal lateral collateral ligament (LCL) sign, and femorotibial rotation (FTR) angle were compared preoperatively and postoperatively. The correlation between the changes in preoperative–postoperative measurements and postoperative measurements with clinical scores and isokinetic measurements was examined. The clinical outcomes were compared based on the presence of a postoperative coronal LCL sign. Inclusion criteria were set as follows: the time between the ACL rupture and surgery being 6 months, availability of preoperative and postoperative clinical scores, and objective determination of muscle strength using isokinetic dynamometer device measurements. Patients with a history of previous knee surgery, additional ligament injuries other than the ACL, evidence of osteoarthritis on direct radiographs, cartilage injuries lower limb deformities, and contralateral knee injuries were excluded from this study. *Results:* This study included 32 patients. After ACL reconstruction, there were no significant changes in the ATT distance (preoperatively: 6.5 ± 3.9 mm, postoperatively: 5.7 ± 3.2 mm) and FTR angle (preoperatively: 5.4° ± 2.9, postoperatively: 5.2° ± 3.5) compared to the preoperative measurements (*p* > 0.05). The clinical measurements were compared based on the presence of a postoperative coronal LCL sign (observed in 17 patients, not observed in 15 patients), and no significant differences were found for all parameters (*p* > 0.05). There were no observed correlations between postoperative FTR angle, postoperative ATT distance, FTR angle change, and ATT distance change values with postoperative clinical scores (*p* > 0.05). Significant correlations were observed between the high strength ratios generated at an angular velocity of 60° and a parameters FTR angle and ATT distance (*p*-values: 0.028, 0.019, and r-values: −0.389, −0.413, respectively). *Conclusions:* Despite undergoing ACL reconstruction, no significant changes were observed in the indirect MRI findings (ATT distance, coronal LCL sign, and FTR angle). These results suggest that postoperative residual tibiofemoral rotation and tibial anterior translation may persist; however, they do not seem to have a direct impact on clinical scores. Furthermore, the increase in tibial translation and rotation could potentially negatively affect the flexion torque compared to the extension torque in movements requiring high torque at low angular velocities.

## 1. Introduction

The complex anatomical and functional structure of the anterior cruciate ligament (ACL) restricts the anterior translation and rotation of the tibia relative to the femur. In the case of a rupture or insufficiency, the knee becomes unstable both anteroposteriorly and rotationally [1,2,3,4,5]. In addition to detecting the disruption of the ACL’s integrity in MRI, indirect findings were described to assist in making a diagnosis. These findings are believed to be related to the presence of knee instability [2,6,7,8,9,10,11,12]. The main indirect findings include the anterior tibial translation (ATT) distance (sensitivity: 56–86%, specificity: 80–99%) [12,13,14], lateral femoral notch sign (sensitivity: 3–19%, specificity: 100%) [15,16], coronal lateral collateral ligament (LCL) sign (sensitivity: 88%, specificity: 92%) [10], femorotibial rotation (FTR) angle (sensitivity: 93%, specificity: 80%) [12], Segond fracture, decreased posterior cruciate ligament angle, and bone marrow edema [13,17].

Lachman and pivot shift tests are among the most important clinical tests for diagnosing ACL ruptures [18]. These tests are provocative tests that indicate static instability in the knee. Furthermore, in the presence of ACL insufficiency, the existence of instability can be demonstrated even without applying a provocative torque to the knee [19]. During the MRI imaging, the knee remains motionless and stationary within the coil. In this manner, the presence of static instability in the knee can be detected in the MRI images. Indirect MRI findings, such as the ATT distance, coronal LCL sign, and FTR angle, are believed to be indicative of the anteroposterior and rotational static instability that occurs after an ACL tear [7,8,10,12,13]. These findings are commonly used as supportive diagnostic tests; however, Mitchell et al. [19] have demonstrated that the presence of a coronal LCL sign may increases the risk of failure after ACL reconstruction.

Many studies have been conducted to evaluate both static and dynamic instability by applying provocative torques after ACL reconstruction [20,21,22,23]. In our study, we believe that we can detect static instability using indirect MRI findings, even without applying any torque. Our hypothesis is that after ACL reconstruction, when sufficient stability is achieved, the indirect MRI findings will return to normal. Therefore, the aim of this study was to compare indirect MRI findings, such as the ATT distance, coronal LCL sign, and FTR angle, before and after ACL reconstruction. Furthermore, this study aimed to investigate the correlation between the measurements of these findings after ACL reconstruction and the changes in measurements compared to preoperative values with clinical outcomes.

## 2. Materials and Methods

After obtaining approval from the Institutional Review Board (SÜKAEK-2023 6/17), a retrospective analysis was conducted on patients aged 18 years and older who underwent isolated anterior cruciate ligament reconstruction between December 2019 and December 2021. To standardize this study, patients who underwent ACL reconstruction with the same technique (anatomical reconstruction with adjustable suspensory fixation system) [24], performed by a single surgeon (LK) with over 400 cases of ACL reconstructions experience annually, were included in this study. The other inclusion criteria were set as follows: the time between ACL rupture and surgery being 6 months, availability of preoperative (preop) and postoperative (postop) clinical scores (Lysholm knee scoring scale (LKSS), Tegner activity score (TAS), International Knee Documentation Committee score (IKDC)), and objective determination of muscle strength using isokinetic dynamometer device measurements. Furthermore, the inclusion criteria required that the time between preoperative MRI imaging and the trauma should be 3 months, and postoperative MRI images should be taken at the 6th month after surgery. Patients with a history of previous knee surgery, additional ligament injuries other than ACL, evidence of osteoarthritis on direct radiographs (Kellgren Lawrence stage 3–4), cartilage injuries, lower limb deformities, and contralateral knee injuries were excluded from this study. As a result, a total of 32 patients were included in this study. Demographic data of the patients, including age, gender, and BMI, were retrospectively recorded.

In this study, we compared the preoperative and postoperative (6th month) MRI measurements of indirect findings, namely ATT distance, coronal LCL sign, and FTR angle, which are believed to be related to knee instability. This study investigated the correlation between the changes in preoperative and postoperative measurements and the postoperative measurements with clinical scores and isokinetic measurements. The clinical outcomes were compared based on the presence of a postoperative coronal LCL sign.

The MRI measurements were performed by two orthopaedic specialists (YSK, HA) with at least 10 years of experience, without knowledge of the clinical findings. The measurements were independently performed for all the findings. In order to assess interobserver reliability, additional measurements were performed on randomly selected 20 MRI images by the observers. Interclass correlation coefficients (ICC) were calculated based on the measurements. The ICC values for the postop coronal LCL sign, postop ATT distance, and postop FTR angle were calculated as 0.862, 0.788, and 0.914, respectively, and the level of interobserver reliability was found to be satisfactory.

### 2.1. MRI Evaluation Methods

The measurements were performed using the Picture Archiving and Communication System (PACS) of our hospital’s diagnostic imaging centre. The patients’ MR images were obtained using a 1.5 Tesla MRI scanner (Royal Philips, Amsterdam, Netherlands) with the knee in 10–15 degrees of flexion. The MRI images were obtained using the knee coil in a position that the patient found most comfortable, without applying any rotational stress to the knee.

**Figure 1:**
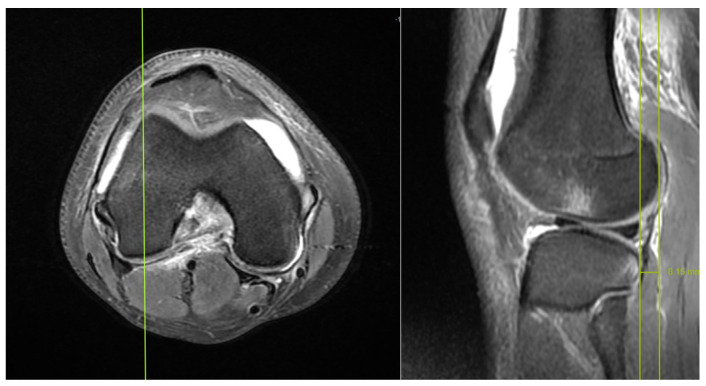
Measurements were taken along the sagittal section passing through the midpoint of the lateral femoral condyle in an axial section. Two parallel lines were drawn tangentially to the posterior cortex of the lateral femoral condyle and the posterior cortex of the tibia in a cephalocaudal axis. The distance between these lines was measured in millimetres (mm) to determine the ATT distance.

The measurement of ATT distance was performed according to the recommended method in the literature [7,13,14]. Measurements were performed by identifying the midpoint of the lateral femoral condyle in the axial section, and then by measuring along the sagittal section passing through this point. Two parallel lines were drawn on the cephalocaudal axis, tangent to the posterior cortex of the lateral femoral condyle and the posterior cortex of the tibia. The distance between these two lines was measured in millimetres (mm) to determine the ATT distance (Figure 1).

**Figure 2:**
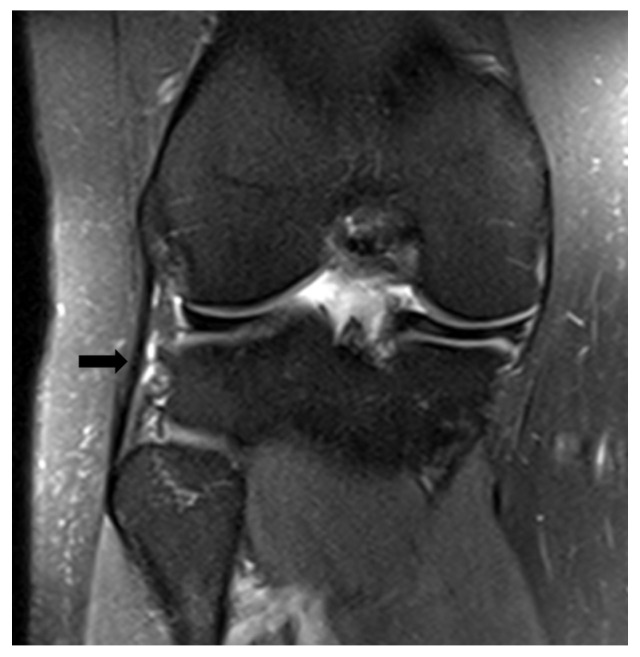
The coronal LCL sign is observed in a single coronal section. The lateral collateral ligament is indicated with an arrow.

The coronal LCL sign was identified as described by Mitchell et al. [8], and a positive sign was recorded when the entire lateral collateral ligament (LCL) was observed in a single MRI coronal section from the fibular head to the lateral femoral epicondyle (Figure 2).

**Figure 3:**
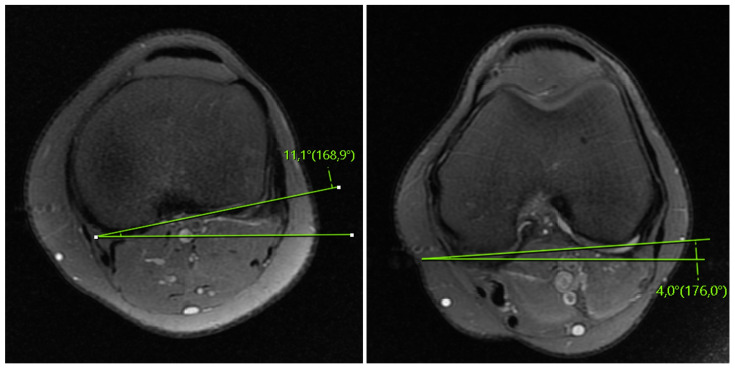
Femorotibial rotation angle evaluation: The femorotibial rotation (FTR) angle was determined as the difference between the tibial angle and the femoral angle. In the above figure, the FTR angle measures 7.1 degrees.

The FTR angle measurement was performed using MRI axial sections, as described by Vassalou et al. [12], and the measurement of femoral rotation was performed using the axial section at the level where the femoral condyles were widest in the anteroposterior direction. The angle between the line tangent to the posterior cortices of the femoral condyles and the horizontal line was determined as the femoral angle. The tibial rotation measurement was performed using the first axial section above the fibular head. The angle between the line tangent to the posterior cortices of the tibial condyles and the horizontal line was considered as the tibial angle. Internal rotation was considered as positive, and external rotation was considered as a negative value. The femorotibial rotation angle was obtained by measuring the absolute difference between these two angles (Figure 3).

### 2.2. Clinical Evaluation

The examination findings of all patients were retrospectively reviewed. All patients included in this study had a positive preoperative Lachman or pivot shift test. Furthermore, all patients had a complete tear of the ACL as documented in the intraoperative notes obtained during arthroscopy.

For a clinical evaluation, the Lysholm knee scoring scale (LKSS), Tegner activity score (TAS), and International Knee Documentation Committee score (IKDC) were used preoperatively and at 6 months postoperatively. The same physiotherapy program was applied to all patients postoperatively.

### 2.3. Isokinetic Strength Measurement

**Figure 4:**
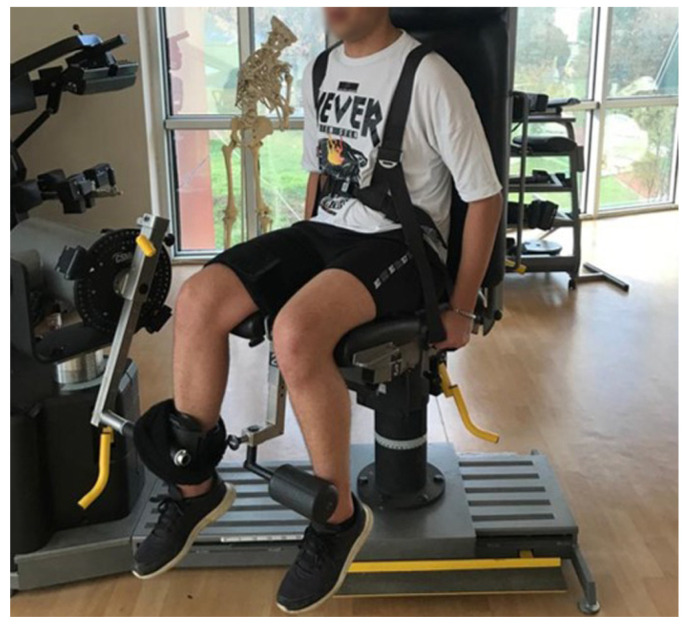
Isokinetic dynamometer device.

At 6 months postoperatively, the isokinetic strength of the knees in the patients with ACL reconstruction was measured. The patients visited the laboratory twice. Before measurements, the belts that prevented body and Q muscle movements were tightened by holding three fingers between the body and the Q muscle, and the subjects held the hand grips on both sides of the chair during the test. During the first visit, a trial measurement was performed for familiarization. On the second visit, the final isokinetic knee strength values were measured. The patients’ knee extension and flexion torques and the hamstring/quadriceps strength ratios were measured using an isokinetic dynamometer device (Humac Norm Testing and Rehabilitation System, CSMI, USA) (Figure 4) at three different angular velocities (60°, 180°, and 240°/s). Measurements for angular velocities of 60°/s and 180°/s were performed with 4 pre-test trials and 5 main test repetitions; for 240°/s, there were 4 pre-test trials followed by 15 main test repetitions. The protocols were applied uniformly to all patients. The torque values obtained from the measurements were recorded in newton meters, and the hamstring/quadriceps (H/Q) ratios were recorded as percentages.

### 2.4. Surgical Technique

The femoral and tibial tunnel drilling procedures are conducted using the anteromedial portal approach. The femoral tunnel placement is positioned between the lateral intercondylar ridge and the posterior cortex of the lateral femoral condyle, leaving a 1–2 mm edge on the femoral condyle. The tibial tunnel is situated within the interspinous distance, aligned with the anterior horn of the lateral meniscus, and approximately 10–15 mm anterior to the anterior border of the posterior cruciate ligament (PCL).

**Figure 5:**
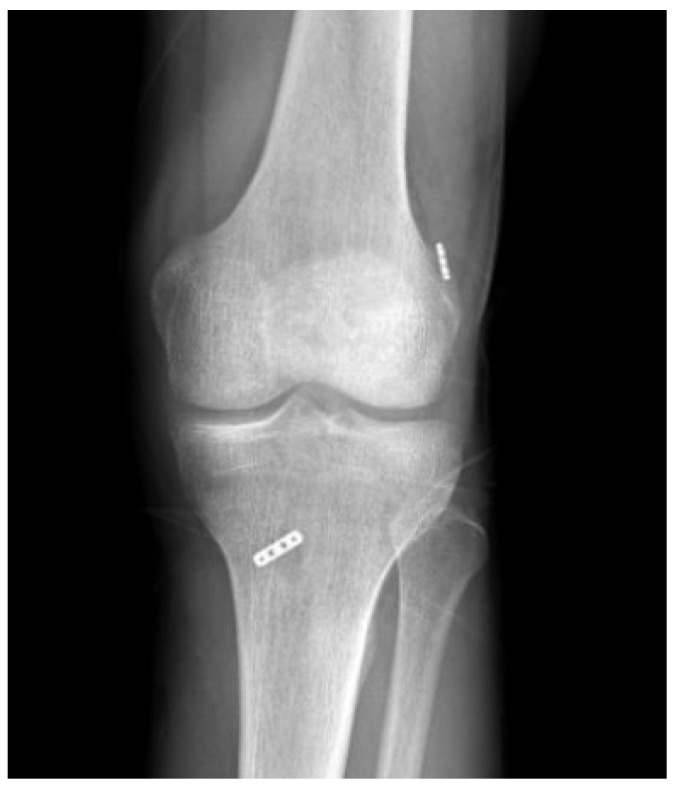
The postoperative radiograph of a patient who underwent anatomical reconstruction using an adjustable suspensory fixation system for ACL reconstruction is presented.

After harvesting the semitendinosus graft, it is folded into four layers and securely affixed to both sides of the suspension fixation loops using No.2 FiberWire sutures. The lengths of the fixation loops are 12 mm on the femoral side and 21 mm on the tibial side. The graft diameters of the included patients range from 8 to 10 mm. For the proper functioning of the suspension fixation system, the graft length is maintained between 6.5 and 7 cm, while tunnel dimensions are adjusted based on the graft diameters. Fixation is achieved through the suspension system with the knee flexed at 30 degrees (Figure 5).

### 2.5. Statistical Analysis

Statistical analysis was performed using SPSS software version 21 (SPSS Inc., IBM, Armonk, NY, USA). Categorical variables were reported as frequencies, and continuous numerical variables were reported as mean and standard deviation values. The homogeneity of variances was examined for continuous variables, and a normality analysis was performed using the Kolmogorov–Smirnov test. For data that followed a normal distribution, paired and independent samples t-tests were used for comparisons. Non-normally distributed data, on the other hand, were analysed using the Mann–Whitney U-test. The correlation between continuous variables was analysed using Spearman’s bivariate correlation test. The agreement between categorical variables was analysed using Cohen’s kappa test, and the reliability between continuous variables was analysed using Cronbach’s alpha reliability test. *p* ≤ 0.05 was considered statistically significant.

## 3. Results

A total of 32 patients were included in this study, consisting of 12 females and 20 males. The demographic data of the patients, preoperative and postoperative knee scores, and differences between preoperative and postoperative measurements are summarized in Table 1.

No significant differences were found between preoperative and postoperative ATT distance and FTR angle MRI measurements (*p*-values: 0.325, 0.506, respectively) (Table 2).

A coronal LCL sign was observed in 17 patients and not observed in 15 patients in the postoperative MRI measurements. In 2 patients who had a coronal LCL sign preoperatively, this sign was not observed postoperatively. On the other hand, in 2 patients who did not have this sign preoperatively, it was observed postoperatively (Table 3). The coronal LCL finding was evaluated in both preop and postop MRI images, and agreement was examined using Cohen’s kappa test (kappa coefficient: 0.749, *p* value < 0.01). In summary, there was a significant agreement between the presence of preop and postop coronal LCL signs (Table 3). Postoperative scores and isokinetic measurements were compared according to the presence of the postoperative coronal LCL sign, and no significant differences were found for all parameters (*p* > 0.05).

The summary of all parameters is presented in Table 4.

Spearman’s correlation analysis was employed to assess the correlation between the postop FTR angle, postop ATT distance, FTR angle change, and ATT distance change values with postoperative scores and isokinetic measurements. The obtained data are presented in Table 5. No significant correlation was found between the postop FTR angle, postop ATT distance, FTR angle change, and ATT distance change values with postoperative scores (*p* > 0.05). A significant negative correlation was observed between the high strength ratios generated at an angular velocity of 60 degrees H/Q ratio and the FTR angle and ATT distance parameters (*p*-values: 0.028, 0.019, respectively; r-values: −0.389, −0.413, respectively). A significant negative correlation was found between the ATT distance change value and the strength ratio (H/Q ratio) at an angular velocity of 240 degrees (*p*-value: 0.018, r-value: −0.416).

Spearman’s correlation analysis was employed to assess the correlation between postop FTR angle, postop ATT distance, FTR angle change, and ATT distance change values with postoperative scores and isokinetic measurements. The obtained data are presented in Table 5. No significant correlation was found between postop FTR angle, postop ATT distance, FTR angle change, and ATT distance change values with postoperative scores (*p* > 0.05). A significant negative correlation was observed between the high torque ratios generated at an angular velocity of 60 degrees H/Q ratio and the FTR angle and ATT distance parameters (*p*-values: 0.028, 0.019, respectively; r-values: −0.389, −0.413, respectively). A significant negative correlation was found between the ATT distance change value and the strength ratio (H/Q ratio) at an angular velocity of 240 degrees (*p*-value: 0.018, r-value: −0.416).

## 4. Discussion

The most significant finding of this study is that the indirect MRI signs thought to be caused by knee instability, such as the ATT distance, Coronal LCL sign, and FTR angle, did not change significantly after ACL reconstruction. The results, which are contrary to our hypothesis, suggest that even after ACL reconstruction, residual rotation and anterior translation may continue to occur in the knee. Although it was observed that postoperative tibial translation and rotation did not correlate with clinical scores, a reverse correlation was found in isokinetic measurements for low-speed, high-torque movements during flexion/extension in terms of strength ratios. The coronal LCL sign is defined as the appearance of the lateral collateral ligament (LCL) as a single coronal section after tibial anterior translation and internal rotation, which is associated with ACL insufficiency, and it is reported to be correlated with ACL ruptures [8]. This finding is present in the literature as a reflection of knee instability [10,25,26,27]. In our study, this finding was evaluated in preoperative and postoperative MRIs, and it was observed that there was no significant change after the surgery in 88.2% of cases, with a substantial level of agreement (kappa coefficient: 0.749, *p* value < 0.01). This result can be initially interpreted as the inability to achieve pre-rupture rotational stability after ACL reconstruction. However, as an opposing view, Hong et al. [26] emphasized in their study that there is no relationship between the coronal LCL sign and instability. In the study conducted by Hong et al., it is emphasized that the pivot shift test was objectively measured using an electromagnetic sensor. In the study conducted by Mitchell et al. [19], it was stated that the presence of the preoperative coronal LCL sign is associated with graft failure; however, it was highlighted that it does not have any effect on clinical scores. Our study demonstrated that the presence of the postoperative coronal LCL sign is not correlated with early clinical outcomes (clinical scores and isokinetic measurements), supporting the findings of this study. Additionally, in our study, two patients who did not have the preoperative coronal LCL sign exhibited this finding postoperatively. To the best of our knowledge, the evaluation of the postoperative coronal LCL sign in MRIs has not been previously reported. Although the exact reason for the emergence of this finding in the postoperative period when it was not present in the preoperative period is not fully understood, we believe that individual anatomical differences may play a role.

The ATT distance represents the translation of the tibia relative to the femur. In the literature, the consensus is that an increase of more than 5 mm in the ATT distance can aid in the diagnosis of an ACL rupture [7,13,28]. Various techniques are used to measure tibial anterior translation; however, the KT-1000 arthrometer is widely used for this purpose [1,18,29,30,31,32,33,34,35]. In their study, which used this device, Papannagari et al. [36] evaluated in vivo knee kinematics after ACL reconstruction. They observed a significant increase in anterior translation in the knee that underwent ACL reconstruction during weight-bearing conditions. In the study conducted by Tashman et al. [37], it was reported that anterior tibial translation increased again between 5 to 12 months after ACL reconstruction. There are publications that suggest the opposite, stating that tibial translation returns to near-normal levels after ACL reconstruction [31,38,39]. In the study conducted by Brandsson et al. [31], anterior laxity was evaluated after ACL reconstruction using radiostereometric analysis and KT-1000 arthrometer measurements, and it was demonstrated that it returned to near-normal levels. However, all these studies have been conducted to evaluate static instability by applying force [35]. In the study conducted by Tagesson et al. [21], it was stated that static tibial translation did not change compared to normal knees after ACL reconstruction; however, they found an increase in dynamic tibial translation. Additionally, it was emphasized that static and dynamic tibial translation are not correlated with each other. Our study, unlike other clinical studies mentioned in the literature, performed tibial translation via MRI after ACL reconstruction without the influence of torque factors. Even though there was no influence of torque factors, we observed residual tibial translation. According to this result, several factors could be influential, including graft selection, the applied rehabilitation program, and the surgical technique used. The study conducted by Hyder et al. [34] emphasized that there is no association between functional outcomes and arthrometric measurements after ACL reconstruction. Although there may be opposing views, numerous previous studies have highlighted that there are no correlations between clinical outcomes and arthrometric measurements [31,33,40]. The difference in the measurement technique that were used between our study and the previous studies could also affect the results. One of the findings of our study is that the presence of residual anterior tibial translation does not reflect on early clinical scores. Moreover, it was observed that the change in tibial translation between preoperative and postoperative periods did not affect the clinical scores. However, in our study, it was observed that tibial translation was not correlated with low angular velocity isolated torques; however, it showed a negative correlation with strength ratios (60° H/Q ratio). The increase or no change in tibial translation can be interpreted as a weaker flexion torque compared to the extension torque in movements requiring low angular velocity and high torque. In addition, a reverse correlation was found between the change in tibial translation (preoperative to postoperative) and high angular velocity strength ratios (240° H/Q ratio). The significant decrease in tibial translation after the surgery was interpreted as a potential weakness of the flexion torque compared to the extension torque in movements requiring high angular velocity. The persistence of residual instability can influence lever arms, which, in turn, may affect strength ratios. Furthermore, in our study, the use of a hamstring (quadruple semitendinosus) graft might be considered to influence strength ratios.

The increase in the femorotibial rotation angle is one of the indirect MRI findings used in the diagnosis of ACL ruptures [12,41,42]. In the study conducted by Vassalou et al. [12], they emphasized that the increased FTR angle on MRI was helpful in diagnosing ACL ruptures. In our study, we hypothesized that there would be a decrease in the FTR angle after ACL reconstruction when sufficient rotational stability is achieved. However, we did not find a statistically significant decrease in the FTR angle. We interpreted this result as the continuation of residual rotational instability after ACL reconstruction. The absence of ALL reconstruction or lateral extra-articular tenodesis in the patients included in our study could be among the reasons for the residual rotational instability. In a systematic review conducted by Zee et al. [42] to evaluate the effect of ACL reconstruction on tibial rotation, 13 studies were analysed. The review highlighted that tibial rotation decreased by approximately 17–32% after ACL reconstruction; however, due to variations in measurement methods, it remains uncertain whether the knee returns to pre-injury levels. In the study conducted by Lian et al. [43], they emphasized the presence of rotational instability even in cases of partial ACL injuries. Tashman et al. [40] conducted a study where they emphasized that ACL reconstruction did not restore normal rotational knee kinematics after dynamic loading. The presence of residual rotational instability after ACL reconstruction in our study supports previous research findings. One of the significant findings of our study is the lack of correlation between postoperative FTR angle measurements and early clinical scores. Even in the presence of postoperative residual rotational instability, it may not affect early clinical scores. However, similar to tibial translational measurements in isokinetic assessments, postoperative FTR angle measurements also showed a negative correlation with strength ratios (60° H/Q ratio) during low angular velocity movements. The increase in tibial rotation was interpreted as a weaker flexion torque compared to the extension torque during movements that require low angular velocity and high torque.

The absence of significant changes in postoperative translation and rotation indicates that residual instability may persist even after surgical intervention. In addition, it was determined that the presence of residual instability was not reflected in the early clinical results. While we might not fully elucidate this outcome, among other reasons, the postoperative state of residual instability being of minimal and static nature can be considered. Consequently, the dynamic process initiated by the movement of surrounding muscle tissues might have obscured the static instability of the knee. This situation supports the conclusion that there is no pronounced reflection on early stage clinical outcomes. However, the prolonged persistence of static instability could potentially lead to various issues in the knee during later stages, thereby influencing potential clinical outcomes. We believe that future studies will shed more light on this matter.

One notable difference of our study from other research is that we conducted measurements using MRIs after ACL reconstruction. To the best of our knowledge, our study is the first to evaluate indirect MRI findings after ACL reconstruction. Our study differs from previous research, as we assessed static instability without applying torque by conducting measurements on postoperative MRI scans. In order to enhance the objectivity of our clinical outcomes, we included isokinetic dynamometer device measurements in addition to subjective knee scores in our study. Our study has natural limitations due to its retrospective design. In addition, none of the patients included in this study underwent ALL reconstruction or lateral extra-articular tenodesis. The evaluation of postoperative LCL findings and rotational instability may yield different results in cases where these procedures are performed. The use of a hamstring (quadruple semitendinosus) graft in our study might have influenced the isokinetic strength results. Furthermore, the use of a hamstring tendon graft might also have an impact on the FTR angle and ATT distance. Future research involving different graft methods should provide a more comprehensive understanding of the relationship between the FTR angle and ATT distance measurements and the strength of the hamstring and quadriceps. Furthermore, the limited sample size in our study constitutes one of the significant limitations of this research. Moreover, due to the absence of MRI images for the healthy knees of our patients, it was not possible to determine the extent to which the postoperative measurements approached the individuals’ own normal values. Additionally, in our study, isokinetic measurements were conducted at 6 months postoperatively, reflecting early term outcomes. Other limitations of this study include the lack of evaluation of tunnel placements.

## 5. Conclusions

Despite undergoing ACL reconstruction, no significant changes were observed in the indirect MRI findings (ATT distance, coronal LCL sign, and FTR angle). These results suggest that postoperative residual tibiofemoral rotation and tibial anterior translation may persist; however, they do not seem to have a direct impact on clinical scores. Furthermore, the increase in tibial translation and rotation could potentially negatively affect the flexion torque compared to the extension torque in movements requiring high torque at low angular velocities.

## Figures and Tables

**Table 1:** Demographic and clinical characteristics, magnetic resonance imaging measurements, and knee scores before and after surgery.

	*n*	Mean	Std. Deviation
Age (years)	32	25.72	6.55
Weight (kg)	32	77.06	9.84
Height (m)	32	176.47	6.95
Body Mass Index (kg/m2)	32	24.71	2.32
Preop Lysholm Knee Scoring Scale	32	72.25	8.28
Postop Lysholm Knee Scoring Score	32	98.59	2.47
Preop IKDC Score	32	49.88	8.32
Postop IKDC Score	32	90.31	5.56
Preop Tegner Activity Score	32	6.38	1
Postop Tegner Activity Score	32	5.91	1.05
ATT Distance Change	32	0.82	3.21
FTR Angle Change	32	0.18	2.8

FTR, femorotibial rotation; ATT, anterior tibial translation; IKDC, International Knee Documentation Committee score; Preop, preoperative; Postop, postoperative.

**Table 2:** Comparison of anterior tibial translation and femorotibial angle before and after surgery.

		*n*	Mean	Std. Deviation	*p* Value
ATT Distance	Preop	32	6.55	3.9	0.325
	Postop	32	5.73	3.2	
FTR Angle	Preop	32	5.45	2.9	0.506
	Postop	32	5.26	3.51	

FTR, femorotibial rotation; ATT, anterior tibial translation; IKDC, International Knee Documentation Committee score; Preop, preoperative; Postop, postoperative.

**Table 3:** Coronal LCL sign, before and after surgery.

		Postop LCL Sign		Total
		No	Yes	
Preop LCL Sign	No	13	2	15
	Yes	2	15	17
Total		15	17	32

Preop, preoperative; Postop, postoperative; LCL, lateral collateral ligament.

**Table 4:** Comparison of postoperative scores and operated side isokinetic measurements according to the presence of postoperative coronal LCL sign.

	Postop Coronal LCL Sign	n	Mean	Std. Deviation	*p* Value
Postop LKSS	Yes	17	99.29	1.64	0.134
	No	15	97.80	3.02	
Postop TAS	Yes	17	5.94	1.19	0.846
	No	15	5.87	0.91	
Postop IKDC Score	Yes	17	91.29	5.61	0.296
	No	15	89.20	5.49	
60°/s Ex	Yes	17	0.82	0.21	0.336
	No	15	0.93	0.26	
60°/s Flx	Yes	17	0.90	0.24	0.209
	No	15	1.00	0.20	
H/Q Ratio 60°/s	Yes	17	1.12	0.30	0.891
	No	15	1.14	0.39	
180°/s Ex	Yes	17	0.84	0.22	0.985
	No	15	0.88	0.20	
180°/s Flx	Yes	17	0.93	0.32	0.760
	No	15	0.96	0.21	
H/Q Raito 180°/s	Yes	17	1.11	0.27	0.865
	No	15	1.15	0.44	
240°/s Ex	Yes	17	0.88	0.21	0.664
	No	15	0.98	0.22	
240°/s Flx	Yes	17	0.95	0.27	0.927
	No	15	0.94	0.18	
H/Q Ratio 240°/s	Yes	17	1.11	0.19	0.199
	No	15	1.00	0.26	

Postop, postoperative; LCL, lateral collateral ligament; IKDC, International Knee Documentation Committee score; LKSS, Lysholm knee scoring scale; TAS, Tegner activity score; Ex, extension; Flx, flexion; H/Q, hamstring/quadriceps.

**Table 5:** The correlate of postop FTR angle, postop ATT distance, FTR angle change, and ATT distance change values with postoperative scores and isokinetic measurements.

					Operated Side Isokinetic Measurements						Operated Side H/Q		
		Postop LKSS	Postop IKDC	Postop TAS	60°/s Ex	60°/s Flx	180°/s Ex	180°/s Flx	240°/s Ex	240°/s Flx	60°/s	180°/s	240°/s
Postop FTR Angle	Correlation Co.	0.133	0.253	−0.121	0.031	−0.158	0.072	−0.123	0.020	−0.079	−0.389 *	−0.324	−0.147
	*p* value	0.235	0.081	0.255	0.868	0.388	0.697	0.502	0.913	0.666	0.028	0.071	0.421
Postop ATT Distance	Correlation Co.	0.196	0.078	−0.051	−0.026	−0.219	−0.127	−0.298	−0.078	−0.267	−0.413 *	−0.132	−0.260
	*p* value	0.142	0.336	0.390	0.887	0.229	0.487	0.097	0.672	0.140	0.019	0.470	0.151
FTR Angle Change	Correlation Co.	−0.246	0.012	0.176	−0.126	−0.228	−0.076	−0.317	−0.121	−0.324	−0.079	−0.174	−0.20
	*p* value	0.087	0.473	0.168	0.493	0.208	0.681	0.077	0.508	0.070	0.667	0.340	0.074
ATT Distance Change	Correlation Co.	0.038	0.010	0.163	−0.197	−0.254	−0.058	−0.278	−0.079	−0.269	−0.026	−0.171	−0.416 *
	*p* value	0.419	0.479	0.186	0.281	0.161	0.751	0.123	0.668	0.136	0.887	0.348	0.018

* *p* < 0.05; Preop, preoperative; Postop, postoperative; IKDC, International Knee Documentation Committee score; LKSS, Lysholm knee scoring scale; TAS, Tegner activity score; Ex, extension; Flx, flexion; H/Q, hamstring/quadriceps; FTR, femorotibial rotation; ATT, anterior tibial translation.

## Data Availability

The dataset that was analysed and generated during this study can be requested from the corresponding authors or yavuzselimkaratekin@gmail.com, as required, in an appropriate manner.
